# College and Hospital Notes

**Published:** 1901-08

**Authors:** 


### COLLEGE AND HOSPITAL NOTES.

The medical department building of the University of Buffalo is to be enlarged by an additional story. It is announced that work will be begun at once under the supervision of George Cary, architect. The new story is to be used for laboratory purposes. The college amphitheater is also to be raised and improved.

The Fresh Air Mission Hospital at Athol Springs affords admirable facilities for the care and treatment of sick babies. It contains thirty beds, has four competent trained nurses, two resident physicians and receives daily visits from the visiting staff. The hospital is free and there is no difficulty to secure admission. Anyone who goes to the Fitch Institute, No. 165 Swan Street, with a card from a reputable physician, or even without a card, will be sent out at once if there is room in the hospital. The hospital is intended especially for cholera infantum and diarrheal diseases, but when there is room receives any patient, infant or adult, who will be benefited. It is most beautifully situated directly on the beach and if babies are not sent too late, a cure is almost assured.

Governor Benjamin B. Odell, Jr., has been spending a few days in Buffalo and vicinity, inspecting the state hospitals and other public institutions. He was accompanied by several state officials, including the chairmen of the appropriation committees of the legislature, and we have no doubt the information secured during this tour of inspection will prove of valuable interest to the commonwealth.

Governor Odell is a business man and applies business methods to his work—an example that might prove of value to some of the officials of our municipal administration. He rises at 6, breakfasts at 7, and by 8 o'clock is well under way for the business of the day. He reached the Buffalo State Hospital by 9 o'clock and gave it one of the most thorough inspections it ever received at the hands of a nonprofessional state official.

Happily, Dr. Hurd's efficient management of the hospital left little for criticism at the hands of the governor; but we have no doubt his visit will lead to still further administrative and economic reforms in all the state institutions.

---

THE Albany Hospital began its series of clinical days, referred to in the July issue of this journal, on Tuesday, July 2, 1901, at which there were between 75 and 100 physicians in attendance from different portions of the country. Dr. Albert Vander Veer delivered the introductory address, taking for his subject The development of medicine and surgery in the Albany Hospital. It was an interesting historical review, beginning with the founders and carrying it down to the present and including the work of such men as Alden March, Thomas Hun, Hoff, S. Oakley Vanderpoel, Henry March, S. H. Freeman, Mason F. Cogswell, James McNaughton, Charles Smith, James P. Boyd, U. G. Bigelow, J. R. Boulware, Joseph Lewi, Jacob S. Mosher, John V. Lansing, James E. Pomfret, W. G. Macdonald, James H. Armsby and others.

The remainder of the program consisted of the following: (1) Amputations at the knee and operation; (2) general sarcomatosis, demonstration of case and treatment; (3) actinomycosis, intraäbdominal, discussion of diagnosis and treatment, two cases; (4) the physical examination of the abdomen. Luncheon at Albany Club; 2.30 p.m., Bender laboratory, general remarks on the use of the microscope, method of examination for the tubercle bacillus, by George Blumer; 3.30 p.m., clinical lecture on chronic nephritis, by Samuel B. Ward. The clinics will be continued during the month of August.

